# Mastering your fellowship: Part 1, 2024

**DOI:** 10.4102/safp.v66i1.5829

**Published:** 2024-01-04

**Authors:** Klaus B. von Pressentin, Mergan Naidoo, Frederick Mayanja, Selvandran C. Rangiah, Ramprakash Kaswa

**Affiliations:** 1 Division of Family Medicine, Department of Family, Community and Emergency Care, Faculty of Health Sciences, University of Cape Town, Cape Town, South Africa; 2 Department of Family Medicine, University of KwaZulu-Natal, Durban, South Africa; 3 Department of Family Medicine and Rural Health, Walter Sisulu University, Mthatha, South Africa

**Keywords:** family physicians, FCFP (SA) examination, family medicine registrars, postgraduate training, national exit examination, infectious diseases

## Abstract

The series, ‘Mastering your Fellowship’, provides examples of the question formats encountered in the written and clinical examinations, Part A of the Fellowship of the College of Family Physicians (South Africa) (FCFP [SA]) examination. The series is aimed at helping family medicine registrars (and their supervisors) in preparing for this examination.

## Introduction

This section in the *South African Family Practice* journal is aimed at helping registrars prepare for the Fellowship of the College of Family Physicians (South Africa) (FCFP [SA]) Final Part A examination and will provide examples of the question formats encountered in the written examination: multiple choice question (MCQ) in the form of single best answer (SBA – Type A) and/or extended matching question (EMQ – Type R); short answer question (SAQ), questions based on the critical reading of a journal article (CRJ: evidence-based medicine) and an example of an objectively structured clinical examination (OSCE) question. Each of these question types is presented based on the College of Family Physicians blueprint and the key learning outcomes (LO) of the FCFP (SA) programme. The MCQs are based on the 10 clinical domains of family medicine, the SAQs are aligned with the five national unit standards and the critical reading section will include evidence-based medicine and primary care research methods.

This edition is based on unit standard one (effectively manage themselves, their team and their practice, in any sector, with visionary leadership and self-awareness, to ensure the provision of high-quality, evidence-based care), unit standard two (evaluate and manage patients with both undifferentiated and more specific problems cost-effectively according to the bio-psycho-social approach), unit standard four (facilitate the learning of others regarding the discipline of family medicine, primary health care and other health-related matters) and unit standard five (conduct all aspects of healthcare in an ethical and professional manner). The clinical domain covered in this edition is infectious diseases. We suggest you attempt to answer the questions (by yourself or with peers or supervisors) before finding the model answers online: http://www.safpj.co.za/.

Please visit the Colleges of Medicine website for guidelines on the Fellowship examination: https://www.cmsa.co.za/view_exam.aspx?QualificationID=9.

We are keen to hear about how this series assists registrars and their supervisors in preparing for the FCFP (SA) examination. Please email us (editor@safpj.co.za) with your feedback and suggestions.

## Extended matching question (EMQ)

*Theme: HIV and Syphilis testing in pregnancy*

**Options:**

- No testing needed
- Rapid syphilis test
- Rapid HIV test
- Dual HIV and syphilis rapid test
- Rapid HIV and rapid plasma reagent (RPR) tests
- ELISA test for HIV and RPR test
- Rapid HIV test and fluorescent treponemal antibody absorption (FTA-ABS) test

For each patient scenario below, match the most appropriate test(s) from the options above. Each option may be used once, more than once or not used.

**Scenarios:**

1. A 25-year-old Para 1 Gravida 2 (P1G2) presents for her third antenatal visit at 30 weeks gestation. Her booking visit at 20 weeks revealed that she had no medical (past or present) problems, and her rapid HIV test and syphilis test were negative. She is asymptomatic, and her vital signs and examination are normal at this visit.
2. A 30-year-old P2G3 presents at 26 weeks gestation. Her booking visit at 18 weeks was unremarkable, and the midwife noted that she was treated for syphilis in her previous pregnancy. She is HIV-negative, had no medical problems and is currently asymptomatic. Her vital signs and examination at this visit are normal.

*Short answer:*

Scenario 1: d

Scenario 2: e

*Discussion:*

**FIGURE 1:**
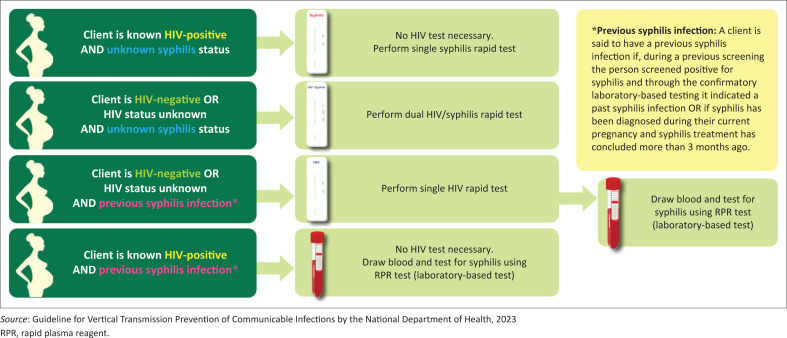
HIV & syphilis testing guide for pregnancy: Which test should be used when?

The recently published 2023 *Guideline for Vertical Transmission Prevention of Communicable Infections* by the National Department of Health has updated the HIV and syphilis testing guidelines during pregnancy, given the adverse clinical outcomes associated with both these conditions. Testing for HIV-negative pregnant women is now recommended at four weekly intervals based on the Basic Antenatal Care Plus (BANC+) visits. Testing occurs at the booking visit and then at 20, 26, 30, 34 and 38 weeks. To improve the efficiency of this new recommendation, a few new point-of-care tests have been introduced. These include the rapid syphilis test, a specific treponemal test that will remain positive for life in a previously infected individual. The dual test incorporates the traditionally used HIV rapid test and the rapid syphilis test. Suppose no positive history of syphilis is provided. In that case, treatment can be initiated for syphilis based on the rapid test, and a confirmatory non-specific RPR test needs to be done (see Figure 1).

**FIGURE 2:**
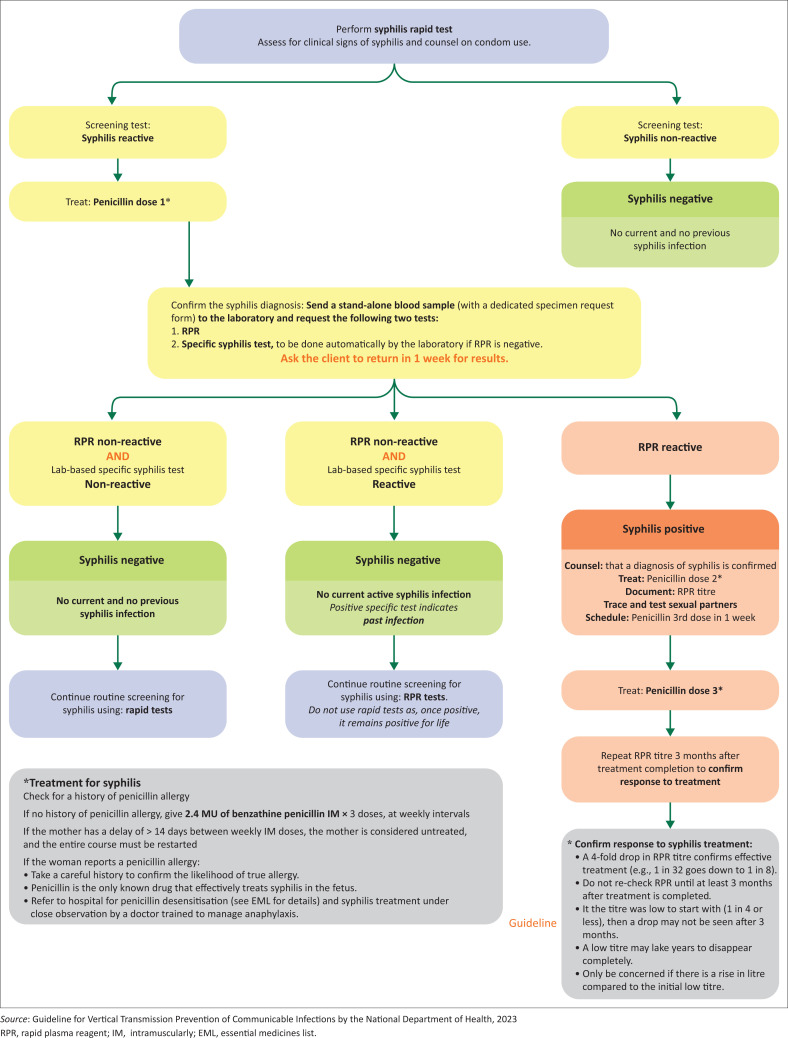
Syphilis rapid diagnostic testing and treatment.

If the facility has no rapid syphilis test, the recommendation is to draw blood for an RPR test during the four weekly visits. Figure 2 outlines the treatment algorithm for patients who test positive for syphilis with the rapid test.

**FIGURE 3:**
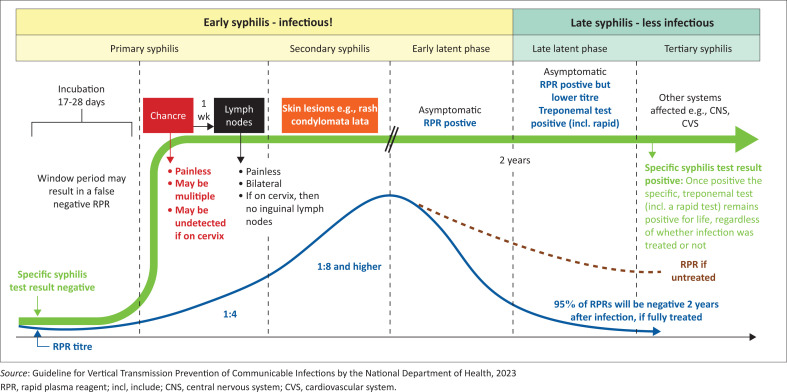
Syphilis testing in primary, secondary and tertiary syphilis.

The patient may be asymptomatic in primary, secondary or tertiary syphilis, so point-of-care testing provides a valuable adjunct to the clinical decision-making process. Figure 3 describes testing related to primary, secondary and tertiary syphilis.

Back to our scenarios: In scenario one, the patient requires routine testing for HIV and syphilis and has no history of previous syphilis, so the dual test is most appropriate and cost-effective as a single point-of-care test will test for both conditions. In scenario 2, the patient gives a history of previous syphilis, so the point-of-care rapid tests are unreliable and the RPR test must be used for the screening process and the titre measured. The RPR is sent to the laboratory, and turnaround time varies based on context. It is important to note that a confirmatory RPR test must accompany a positive rapid test, but the patient must be treated with the first dose of penicillin. Benzathine penicillin is the most effective treatment for syphilis, and three doses are needed in pregnant women.

**Further reading**

- South African National Department of Health. Guideline for vertical transmission prevention of communicable infections. Pretoria: South African National Department of Health; 2023.

## Short answer question (SAQ): The family physicians as a capacity builder, role model, teacher, trainer, supervisor, in the domain of ‘infectious diseases – HIV/TB/malaria’

*This question was previously used in an FCFP (SA) written paper.*

You are the district family physician in Mopane District in Limpopo province of South Africa, where malaria is endemic. A newly employed medical officer (MO) phones you about a 19-year-old male high school student with a fever and impaired consciousness. Other systems are unremarkable. The MO suspects malaria as a coronavirus disease 2019 (COVID-19) test, and a lumbar puncture is negative. The patient is admitted, and the MO asks for further assistance with the assessment and management of the patient. You advise him to do a malaria rapid diagnostic test, which is positive for malaria, and the patient is started on treatment for severe malaria:

1. You realise that junior doctors in the district need more knowledge of malaria. How would you apply the steps of planning and implementing a teaching session on managing severe malaria by doctors at the district hospital level? (8 marks)
2. Provide two learning outcomes (LOs) in the correct format for your teaching session. (2 marks)
3. At the end of the session, you decide to assess the doctors’ ability to manage patients presenting acutely with complicated malaria. List four assessment methods that allow the learner to demonstrate – knows, knows how, shows how, does. (4 marks)
4. What three adult learning principles would you apply, and explain in more detail how you would do this? (6 marks)

**Total: 20 marks**

Suggested answers (the answers should show some application to the scenario)

**1. You realise that junior doctors in the district need more knowledge of malaria. How would you apply the steps of planning and implementing a teaching session on managing severe malaria by doctors at the district hospital level? (8 marks)**

- Step A. Why: (decide the need and objectives) (2 marks)
  ■ Confirm that the topic of malaria is important to address for district health services.
  ■ Clarify the learning needs of the doctors and gaps in knowledge.
- Step B. What: (LOs and content) (2 marks)
  ■ Define LOs for the teaching session, including – knowledge, skills and attitudes. At the end of this session, the learner is expected to classify malaria into uncomplicated and complicated (knowledge) and list the management strategies appropriate to the district hospital level. The learner must outline their knowledge of the drugs and when to use them.
  ■ Define the content and resources needed – for example, the National Institute For Communicable Diseases of South Africa (NICD) national guidelines.
- Step C. How: (teaching methods and logistics) (2 marks)
  ■ Plan the teaching method to be used for example, a PowerPoint lecture followed by small group discussion using case scenarios as part of the hospital’s continuing professional development (CPD) programme.
  ■ Plan logistics: date, venue, equipment needed, CPD points and invitations.
- Step D. So what: (evaluation) (2 marks)
  ■ Obtain feedback from participants – simple questionnaire.
  ■ Reflect on feedback to revise future teaching sessions.

**2. Provide two learning outcomes in the correct format for your teaching session. (2 marks)**

A LO should specify what the doctor can do at the end of the teaching session. The LO could be based on knowledge, skills or attitudes. One mark each for the LO and description of what should be known or done, for example, maximum of two examples for 2 marks in total:

- Knowledge: At the end of this session, you should be able to list the differences between uncomplicated and complicated malaria. (1 mark)
- Skill: At the end of this session, you should be able to demonstrate how to use a rapid malaria test correctly or some aspect of inpatient care and monitoring. (1 mark)
- Attitude: At the end of this session, you should be able to counsel uncomplicated malaria patients on correct drug use and safety netting for when to return to the clinic or hospital. (1 mark)

**3. At the end of the session, you decide to assess the doctors’ ability to manage patients presenting acutely with complicated malaria.** List four assessment methods that allow the learner to demonstrate – knows, knows how, shows how, does. (4 marks)

- Knows – written, oral or computer-based test on knowledge of complicated malaria at the factual recall level. (1 mark)
- Knows how – written, oral or computer-based tests on applying knowledge to a patient case vignette. (1 mark)
- Shows how – demonstrates skill in a simulated setting, for example, simulation scenario of a patient with complicated malaria. (1 mark)
- Does – observed consultations in the emergency room or workplace-based assessment (WPBA), chart review, audit of practice. (1 mark)

**4. What three adult learning principles would you apply and explain in more detail how you would do this? (6 marks)**

*(Any 3 points from the list below, one mark for principle and one mark for how it will be applied)*

- Provide a safe and supportive environment – personal freedom and individuality should be honoured, non-judgemental and trust established.
- Provide an environment that promotes intellectual freedom – learners are allowed to experiment and be creative, with different learning styles.
- Provide an environment that treats learners as peers – learners are respected as intelligent and experienced adults, identify gaps and choose the best learning activities to address these.
- Encourage self-directed learning – learners need to take responsibility for their needs and become actively involved in their learning, as interaction, experimentation and dialogue help cement learnt facts and theory.
- Challenge people beyond their current level of ability – need to establish objectives and learning needs.
- Learners should be actively involved in learning – adopt styles other than didactic lecturing.
- Provide regular feedback to learners by summarising, identifying of future needs, facilitating reflection and helping to develop action plans; these actions ensure constant improvement in a learner-centred way.

**Further reading**

- South African National Department of Health. National guidelines for the treatment of malaria, South Africa [hompage on the Internet]. Pretoria: National Department of Health; 2019 [cited 2023 Jul 18]. Available from: https://knowledgehub.health.gov.za/elibrary/national-guidelines-treatment-malaria-2019
- Brits H. How to plan and implement a teaching activity. In: Mash B, Brits H, Naidoo M, Ras T, editors. SA family practice manual. Cape Town: Van Schaik, 2023; p. 735–742.
- Mehay R, editor. The essential handbook for GP training and education. London: CRC Press, 2013; London; p. 114–117.

## Critical appraisal of quantitative research

Read the accompanying article carefully and then answer the following questions. As far as possible use your own words. Do not copy out chunks from the article. Be guided by the allocation of marks concerning the length of your responses.

- Hoque M, Hoque ME, Van Hal G, Buckus S. Prevalence, incidence and seroconversion of HIV and syphilis infections among pregnant women of South Africa. S Afr J Infect Dis. 2021;36(1):a296. https://doi.org/10.4102/sajid.v36i1.296

**Total: 30 marks**

1. Critically appraise the authors’ choice of a retrospective cohort study for addressing the research question. (4 marks)
2. Critically appraise the sampling strategy. (4 marks)
3. Describe aspects related to the ethical considerations for conducting this study. (4 marks)
4. Critically appraise the choice of the statistical tests considered for this study. (5 marks)
5. Did the study manage to address a focused research question? Discuss. (3 marks)
6. Critically analyse the limitations of the study. (4 marks)
7. Use a structured approach (e.g., relevance, education, applicability, discrimination, evaluation, reaction [READER]) to discuss the value of these findings to your practice. (6 marks)

*Suggested answers:*

### 1. Critically appraise the authors’ choice of a retrospective cohort study for addressing the research question. (4 marks)

- A cohort study is a quantitative study that aims to determine a condition’s natural history and incidence. It uses a longitudinal design to analyse the progression of the disease. In addition, it can calculate the hazard ratio, incidence rate, relative risk and cumulative incidence.
- Although it is not always possible to establish causality in a study, cohort studies can provide valuable information on the link between various risk factors and outcomes. This process involves assessing the effects of exposure on a group of subjects who were not exposed to a certain factor.
- Unlike cross-sectional studies, cohort studies are not used to determine prevalence. Instead, they are used to study causes and incidence.
- In this study, the retrospective cohort study design represented a time-efficient approach to address the study objectives, and data are readily available from existing patient records in the clinic register.

### 2. Critically appraise the sampling strategy. (4 marks)

- The researchers chose a peri-urban primary healthcare setup that provides first-level care to predominantly Zulu-speaking black African populations. Members of the research team were based at this facility.
- No sampling was done, as the researchers collected data from the antenatal clinic register of all pregnant women who attended booking visits between January 2018 and December 2018. No rationale for this time interval was provided. One calendar year is a convenient choice but may not reflect the trends over a longer period. It may also be useful to reflect on how basic antenatal care (BANC) protocols may have varied over this period. Some would question whether 1 year falls into the category of a cross-sectional study.
- Figure 1 shows how the data of the 1503 study subjects were organised during the data analysis. It is important to note that this data only reflects the total number of pregnant women who received all or aspects of their antenatal care experience at this facility during this period and excludes the unbooked patients or patients who received antenatal care elsewhere in the health system (private vs public, district health services vs specialist level care). As such this may not reflect the true prevalence or incidence of the conditions of interest at the community level.
- The researchers did not specify how they addressed any potential biases, which may have been because of the frequency of antenatal visits, including more frequent visits compared to the norm of the antenatal care package and late booking in the antenatal period.

### 3. Describe aspects related to the ethical considerations for conducting this study. (4 marks)

Conducting research is a process that involves making ethical decisions. These principles help guide the design and execution of studies. Researchers must follow a code of conduct when gathering information from individuals. Human research aims to understand real-world phenomena, explore effective treatments, investigate behaviours and explore diverse ways of improving people’s lives. When it comes to conducting studies, ethical considerations play a significant role. These considerations protect the rights of research participants, enhance research validity and keep scientific or academic integrity. The ethical consideration relevant to this study consists of the following aspects:

- Ethics committee review and site access approval: The Umgungundlovu Health Ethics Review Board approved the study protocol. The Kwadabeka Community Health Centre’s management provided written permission to allow the use of the antenatal clinic register.
- Informed consent process: The informed consent was waived because there was no direct contact with participants. No new data were generated as the researchers used secondary data from the register containing data collected as part of routine care.
- Protection of personal information: The researchers maintained strict privacy and confidentiality during the study.
- Prevention of third-party injury: There was no statement about all other forms of harm, including psychological, social and physical as the researchers accessed the patients’ identity and diagnosis via the clinic register. However, this retrospective study design did not involve any experimental interventions and patient data were collected as part of routine clinical care.

### 4. Critically appraise the choice of the statistical tests considered for this study. (5 marks)

The statistical analysis of a cohort study assesses the association between multiple exposures and outcomes over time and builds prognostic or prediction models. In this study, the authors use the following statistical tests:

- Pearson chi-square (χ^2^) and *p*-values
- Regression
- Adjusted odds ratio (OR) with corresponding 95% confidence intervals (95% CI) and *p*-values

- Categorical variables were presented as proportions and frequencies. The differences in the proportions of HIV and syphilis among the different obstetric and demographic elements at the booking visit were then examined using the Pearson chi-square and *p*-values.
- The study used a step-by-step approach to analyse the variables to determine the risk factors for HIV and syphilis incidence and prevalence during pregnancy. The results were then presented with an adjusted OR.
- A step-by-step binary logistic regression procedure was then performed to identify the factors that could influence the prevalence and incidence of both HIV and syphilis.
- The regression results were presented with an adjusted OR of 95% confidence intervals and *p*-values. The *p*-values under 0.05 were regarded as significant.
- In cohort studies, model building is crucial. Researchers may need to develop explanatory models focused on identifying factors that have a significant relationship with an outcome. On the other hand, in predictive models, the goal is to predict an individual’s likelihood of experiencing a future occurrence or a possible diagnosis. The current study did not use any model building for identifying risk factors for HIV and syphilis seroconversion during pregnancy.

### 5. Did the study manage to address a focused research question? Discuss. (3 marks)

- The study aimed to help health officials plan how best to manage and control syphilis and HIV infections among pregnant women. It sought to estimate these conditions’ prevalence and risk factors during antenatal care.
- The research question was focused as it described the population of interest (pregnant women attending antenatal care at the peri-urban primary health centre) and the condition or phenomenon of interest (prevalence, incidence and seroconversion of HIV and syphilis during pregnancy) in a particular community or area (Kwadabeka Community Health Centre of Durban, South Africa).
- The study determined the prevalence, seroconversion and incidence of HIV and syphilis. Furthermore, associated risk factors for syphilis were identified, especially maternal age, parity and HIV status.

### 6. Critically analyse the limitations of the study. (4 marks)

- The retrospective cohort study is limited because of the nature of its research design. The following limitations should be considered:
  ■ Susceptible to loss to follow-up compared with cross-sectional studies.
  ■ Confounding variables are the major problem in analysing the data.
  ■ Susceptible to information bias and recall bias.
  ■ Less control over variables.
- The study lacks sufficient evidence to establish the risk of HIV and syphilis seroconversion in pregnant women who visited the antenatal care (ANC) clinic. There were limited study variables for risk factors. It also did not consider the factors that affect the new infection, such as the availability of resources and the socio-economic status of the women. The study might have underestimated the prevalence of these conditions because of the lack of access to the clinic and the cost of travel.
- The authors have no control over the variables and information bias. The data were collected retrospectively from clinical records. The rates might have underestimated or overestimated the prevalence of syphilis and HIV as many women were missing because of inaccessibility to the ANC clinic.
- Loss to follow-up (syphilis follow-up rate was 77%) and confounding factors during data analysis directly influence the associated risk between HIV and syphilis seroconversion.

### 7. Use a structured approach (e.g., relevance, education, applicability, discrimination, evaluation, reaction [READER]) to discuss the value of these findings to your practice. (6 marks)

The READER format may be used to answer this question:

- Relevance to family medicine and primary care?
- Education – does it challenge existing knowledge or thinking?
- Applicability – are the results applicable to my practice?
- Discrimination – is the study scientifically valid enough?
- Evaluation – given the aforementioned, how would I score or evaluate the usefulness of this study for my practice?
- Reaction – what will I do with the study findings?

*The answer may be a subjective response but should show a reflection on the possible changes within the student’s practice within the South African public healthcare system. It is acceptable for the student to suggest how their practice might change within other scenarios after graduation (e.g., private general practice). The reflection on whether all essential outcomes were considered depends on the reader’s perspective (is there other information you would have liked to see?)*.

*A model answer could be written from the perspective of the family physician employed in the South African district health system:*

- R: This study is relevant to the African primary care context, as HIV and syphilis are common health concerns during pregnancy. A better understanding of the risk factors of HIV and syphilis seroconversion during antenatal care in primary care clinics will help primary care teams, and policymakers plan appropriate interventions.
- E: Among the pregnant women attending ANC in South Africa, the prevalence of syphilis and HIV varies depending on the province; by monitoring the prevalence and incidence of syphilis and HIV among pregnant women, as well as naming risk factors, the author explores the effective interventions to prevent seroconversion during pregnancy. There is inadequate knowledge about the transmission of these diseases among pregnant women in a Midwife-run Obstetric Unit (MOU). This study aims to inform health managers about strategies to control and treat syphilis and HIV infections among pregnant women.
- A: For this study, it would be possible to generalise its findings to a similar South African public sector antenatal clinic.
- D: On discrimination, there is a fair congruity between the research methodology, data collection methods and data analysis. There was a clear lack of inclusion and exclusion criteria for selecting study participants. (It would have been helpful to state the methodological position). Furthermore, the researchers did not describe the comparative cohort group with different characteristics exposure, although the clinical event of interest (HIV and syphilis) was well explained.
- E: The study’s findings may be relevant when supplying antenatal care services. The study findings did point to the high prevalence of HIV and syphilis among pregnant women. Furthermore, the study highlighted the role of primary care providers in the prevention of seroconversion of HIV and syphilis in a high-risk category (pregnancy or antenatal period), as well as the need for counselling and testing, antiretroviral therapy (ART) and health education to change the behaviour on prevention of HIV and syphilis among pregnant women in particular and the general population at large. It may be helpful to remind oneself of the nature of the study design and its limitations and that the study setting-related behavioural factors influence HIV and syphilis seroconversions.
- R: The study’s findings may help manage HIV and syphilis during antenatal care in primary health care settings. The study explained the higher rates of HIV and syphilis infection among pregnant women that lead to adverse perinatal outcomes. Effective interventions, such as testing and counselling, should be implemented in primary care to minimise the impact of these infections on pregnancy outcomes.

**Further reading**

- Pather M. Evidence-based family medicine. In: Mash B, editor. Handbook of family medicine. 4th ed. Cape Town: Oxford University Press, 2017; p. 430–453.
- MacAuley D. READER: An acronym to aid critical reading by general practitioners. Br J Gen Pract. 1994;44(379):83–85.
- The Critical Appraisals Skills Programme (CASP). 2023. CASP checklists [homepage on the Internet]. [cited 2023 Feb 04]. Available from: https://casp-uk.net/casp-tools-checklists/
- Goodyear-Smith F, Mash B, editor. How to do primary care research. Boca Raton, FL: CRC Press, Taylor and Francis Group; 2019.

## Objectively structured clinical examination station scenario (OSCE)

**The objective of the station:** This station tests the candidate’s ability to consult with an HIV-positive patient requesting malaria prophylaxis and yellow fever vaccination for travel purposes.

**Type of station:** Integrated consultation.

**Role player:** Simulated patient: adult male or female.

**Instructions to the candidate:**

- You are the family physician working at the consultant clinic of a large district hospital, and the nurse has asked you to consult with this 32-year-old patient.
- Your task: Please consult with this patient.
- You do not need to examine this patient. All examination findings will be provided on request.

**Instructions for the examiner:**

- This is an integrated consultation station in which the candidate has 15 min.
- Familiarise yourself with the assessor guidelines that detail the required responses expected from the candidate.
- No marks are allocated. In the mark sheet (Table 1), tick off one of the three responses for each competency listed. Make sure you are clear on what the criteria are for judging a candidate’s competence in each area.
- Provide the following information to the candidate when requested: examination findings.
- Please switch off your cell phone.
- Please do not prompt the student.
- Please ensure that the station remains tidy and is reset between candidates.
- The aim is to establish that the candidate has an effective and safe approach to counselling an HIV-positive patient seeking malaria prophylaxis and yellow fever vaccination before visiting a malaria and yellow fever endemic area.
- A working definition of competent performance: the candidate effectively completes the task within the allotted time, in a manner that maintains patient safety, even though the execution may not be efficient and well structured:
  ■ *Not competent:* patient safety is compromised (including ethical-legally) or the task is not completed.
  ■ *Competent*: the task is completed safely and effectively.
  ■ *Good*: in addition to displaying competence, the task is completed efficiently and in an empathic, patient-centred manner (acknowledges patient’s ideas, beliefs, expectations, concerns/fears).

**TABLE 1:** Marking sheet for objectively structured clinical examination station scenario.

Competencies		Candidate’s rating		
		Not competent	Competent	Good
1.	Establishes and maintains a good doctor-patient relationship Comment:	-	-	-
2.	Gathering information: history, examination, investigations Comment:	-	-	-
3.	Clinical reasoning Comment:	-	-	-
4.	Explaining and planning Comment:	-	-	--
5.	Management Comment:	-	-	-

### Guidance for examiners regarding Table 1

**Establishes and maintains a good clinician–patient relationship:**

- The competent candidate is respectful and engages with the patient in a dignified manner. *(Ascertains reason for the consultation and makes the patient feel comfortable while ensuring the ground for confidentiality is set)*
- The good candidate is empathic, compassionate and collaborative, facilitating patient participation in key areas of the consultation.

*(Maintains this throughout the consultation)*

**Gathering information**

- The competent candidate gathers sufficient information to establish a clinical assessment (*Detailed relevant history including current and past medical conditions including HIV status, CD4 count, viral load, medications and risk factors upon which a decision may be made*).
  The good candidate additionally has a structured and holistic approach (*explores the patient’s agenda concerning individual and contextual issues*).

**Clinical reasoning**

- The competent candidate identifies the reason for the consultation (*malaria prophylaxis and need for yellow fever vaccination*) and acknowledges the relevant challenges and dilemmas for this patient (*risks, contraindications*).
- The good candidate has a structured approach to addressing the patient’s agenda (*individual and contextual*) and considers other travel medicine options (*typhoid, diarrhoeal disease, hepatitis)*.

**Explaining and planning**

- The competent candidate uses clear language to explain to the patient and uses strategies to ensure patient understanding.
  *For vaccination purposes, persons with asymptomatic HIV infection and CD4+ cell counts of 200/µL to 500/µL are considered to have limited immune deficits and are generally candidates for immunisation. HIV-positive persons with CD4+ cell counts less than 200/µL or a history of an AIDS-defining illness should not receive live-attenuated viral or bacterial vaccines because of the risk of serious systemic disease and suboptimal response to vaccination. This applies to yellow fever vaccination*.
  *HIV-positive adults are more prone to acquiring malaria and are at an increased risk of severe malaria and death. Malaria can worsen HIV disease progression. Therefore, the prevention of malaria is even more important in these individuals*.
  *Further, drugs used for malaria prophylaxis may interact with antiretroviral drugs and there is a general lack of data on the safety and efficacy of antimalarial regimens in patients taking antiretroviral therapy*.
- The good candidate additionally ensures that the patient is actively involved in decision-making, paying particular attention to knowledge-sharing and empowerment, given the dilemma faced by the patient.
  *Travellers with severe immune compromise, including those with symptomatic HIV infection and AIDS, should be strongly discouraged from travelling to destinations that present an actual risk for yellow fever. If travelling to an area at risk of yellow fever is unavoidable, these travellers should be carefully instructed in methods to avoid mosquito bites and be provided with a vaccination medical waiver*.
  *The exemption letter, signed by the physician, simply states ‘Yellow fever vaccine for “NAME” is medically contraindicated because of the following condition:* [*age, pregnancy, immunocompromised status*]*’. However, international health regulations do not allow an exemption from yellow fever vaccination for travel to a country that has a vaccination requirement for entry, even for medical reasons. Thus, travellers should be warned that some countries may not accept vaccination waiver documents. If the waiver is rejected, the option of deportation might be preferable to receipt of the vaccine at the destination. For countries requiring entry vaccination, travellers must have proof that the vaccine was administered at least 10 days before entry*.

## Management

The competent candidate proposes appropriate intervention.

*Counselling about avoiding mosquito bites (e.g., bed netting, insect repellents, permethrin-impregnated clothing). They should also be prescribed appropriate drugs for malaria prophylaxis*.

*Daily doxycycline. The advantages of doxycycline include low cost and preventive effects against diarrhoea, leptospirosis and* Rickettsia *species infections. Doxycycline’s disadvantages include the need to take it daily, associated photosensitivity, the potential for gastrointestinal upset and the need to take it one day before and four weeks after exposure, or*

*Daily atovaquone-proguanil. Advantages of atovaquone-proguanil include its safety and the need to take it only one day before and seven days after exposure; disadvantages include higher cost, the potential for headache, gastrointestinal upset, insomnia and the need to take it daily and with food*.

*Yellow fever vaccination is not recommended because of the patient’s CD4 count*.

*Typhoid vaccine parenteral and not the live oral vaccine – 5 years since the previous vaccine*

The good candidate additionally discusses the *risks of travel given the patient’s low CD4 count and offers a letter of waiver that is subject to visiting countries’ protocols. The patient is counselled regarding individual and contextual issues*.

*Indicates that Mefloquine is no longer available in South Africa*

**Role player instructions**

Adult male/female. 36-year-old patient.

Opening statement: ‘Doctor, I need to travel to the Democratic Republic of Congo, and I want medication for malaria and a yellow fever vaccination. Can you please help me …’.

Open responses: Freely tell the doctor …

- I previously travelled to Zimbabwe in 2019 and I got vaccinated for typhoid and hepatitis.
- I also got COVID-19 vaccinations.
- I just need malaria prevention medication and the yellow fever vaccine.

Closed responses: Only tell the doctor if she or he brings this up:

- You have no complaints except for a minor sore throat.
- You have been diagnosed with diabetes after contracting COVID-19 in 2021.
- You think your glucose is controlled with Metformin 850 mg three times a day.
- You tested positive for HIV 2 years ago and are on Tenofovir, Lamivudine and Dolutegravir (TLD).
- You have no other chronic conditions and no allergies.
- You never had any surgery.
- You do not smoke cigarettes and do not consume alcohol.

You are a refugee living in South Africa since 2010 and have received permanent residency. You work as an Uber driver having completed over 10 000 trips.

You have a South African wife who is also HIV positive and you have two children aged 4 years and 7 years. They will not travel with you to the DRC.

*Ideas, concerns and expectations:*

You are concerned that malaria medication will affect your ARV medication.

You have heard from the media that malaria is very dangerous and kills you. Worried about the well-being of your wife if anything happens to you.

You need to travel urgently to the DRC to attend your father’s funeral; you will not be able to live with yourself if you do not go as your father was responsible for your upbringing, protection and success in life. You graduated as a teacher in the DRC and had to flee because of the civil war in your country.

You also believe that his soul/spirit will not rest if you do not perform the last rites.

You expect the doctor to provide sound advice and treatment to allow you to attend the funeral.

You also want to know if you should take a break from your ARV medication while travelling.

*Examination findings:*

- Body mass index – 19 kg/m^2^.
- Blood pressure – 118/72 mmHg, heart rate: 86 beats/minute.
- Haemoglobin – 11.5 gm/dL; HbA1c – 8.1%.
- Random blood glucose (HGT) – 5.9 mmol/L.
- Urinalysis – No abnormalities.
- CD4 count 190/µL (6 months ago); viral load result pending.
- Ear, nose and throat (ENT) – Normal except for mild oral thrush.
- Cardio-respiratory systems – No abnormalities.
- Abdomen – No abnormalities.
- Neuro – No abnormalities.

**Further reading**

- South African National Department of Health. National guidelines for the prevention of malaria, South Africa [homepage on the Internet]. Pretoria: National Department of Health; 2018 [cited 2023 Jul 18]. Available from: https://www.nicd.ac.za/wp-content/uploads/2019/03/National-Guidelines-for-prevention-of-Malaria_updated-08012019-1.pdf
- Centers for Disease Control and Prevention (CDC). CDC yellow book 2024: Health information for international travel [homepage on the Internet]. [cited 2023 Jul 18]. Available from: https://wwwnc.cdc.gov/travel/page/yellowbook-home
- Smith DS. Travel medicine and vaccines for HIV-infected travellers. Top Antivir Med. 2012;20(3):111–115.

## Data Availability

Data sharing is not applicable to this article as no new data were created or analysed in this study.

